# F165. OBSTRUCTIVE SLEEP APNOEA IS COMMON IN SCHIZOPHRENIA AND RESPONDS WELL TO TREATMENT – A NOVEL AND PRACTICAL MEANS TO IMPROVE COGNITION AND METABOLIC HEALTH?

**DOI:** 10.1093/schbul/sby017.696

**Published:** 2018-04-01

**Authors:** Cherrie Galletly, Hannah Myles, Andrew Vincent, Nicholas Myles, Robert Adams, Madhu Chandratilleke, Dennis Liu, Jeremy Mercer, Andrew Vakulin, Gary Wittert

**Affiliations:** 1 University of Adelaide; 2 Freemasons Foundation Centre for Men’s Health; 3 Royal Adelaide Hospital; 4 Adelaide Institute for Sleep Health: A Flinders Centre for Research Excellence, Flinders University

## Abstract

**Background:**

Obstructive sleep apnoea (OSA) is characterised by repeated collapse of the upper airway during sleep, causing hypoxia, frequent arousals and disruption to sleep architecture. OSA is more likely in people who are obese, smoke tobacco, and use alcohol and sedating medications – all these factors are more common in schizophrenia. OSA is likely to be underdiagnosed in schizophrenia as symptoms such as non-restorative sleep, depression and daytime somnolence may be attributed to chronic mental illness. OSA in the normal population is associated with cognitive deficits and poor cardiovascular health, both of which are common in schizophrenia, so comorbid OSA in schizophrenia may be exacerbating these problems.

Treatment of OSA with continuous positive airway pressure (CPAP) reduces daytime sleepiness, and improves quality of life, cognitive function, and cardiovascular risk factors. There are no published studies of CPAP treatment of OSA in schizophrenia, so it is not known whether these benefits also occur in the patient population.

**Methods:**

Previous research into OSA in schizophrenia has utilised subjective screening instruments and there are no large studies using polysomnography (PSG), the gold standard method to diagnose OSA. We undertook home sleep studies using polysomnography in 30 people with schizophrenia, treated with clozapine. Participants cooperated well and all studies were of good quality.

We treated 6 participants with severe OSA with CPAP. Treatment adherence was good with mean CPAP usage of of 7.7 hours/night.

**Results:**

We found that 14/30 (40%) of our participants with schizophrenia had OSA and 8/30 (27%) had severe OSA; twice the prevalence of severe OSA in the general population.

After six months CPAP treatment there was significant improvement in cognition, especially verbal memory, working memory and motor skills. Average weight loss was 7.2kg (SD 9k) with a 12mmHg (SD 18) reduction in systolic blood pressure. Normal sleep architecture was restored: on average the percentage of the night spent in restorative slow wave sleep increased from 4.8% to 31.6%, and the percentage in REM sleep from an average of 4.1% to 31.4%. The mean percentage of the night spent in a hypoxic state with oxygen saturation less than 90% reduced from an average of 27.6% to 2%.

**Discussion:**

Improved awareness of the high prevalence of OSA in schizophrenia and access to diagnostic screening by home PSG should ensure this important comorbid condition is not missed. CPAP treatment for OSA in people with schizophrenia is feasible and has the potential to improve both cognition and cardiovascular health, resulting in better functioning and reduced cardiovascular morbidity.

